# Optimizing Risk Communication for Lynch Syndrome: Results of a Randomized Controlled Trial of Visual Arrays for Genetic Testing

**DOI:** 10.3390/cancers18091369

**Published:** 2026-04-25

**Authors:** Jordan N. Keels, Viktoriya Babicheva, Isabella R. McDonald, Jessica K. Witt, Andrew A. Dwyer

**Affiliations:** 1 William F. Connell School of Nursing, Boston College, Chestnut Hill, MA 02467, USA; jordan.keels@bc.edu (J.N.K.); isabella_mcdonald@atriushealth.org (I.R.M.); 2 Research Services, Boston College, Chestnut Hill, MA 02467, USA; babichev@bc.edu; 3 Department of Psychology, Colorado State University, Fort Collins, CO 80521, USA; 4 P50 Massachusetts General Hospital—Harvard Center for Reproductive Medicine, Massachusetts General Hospital, Boston, MA 02114, USA

**Keywords:** colorectal cancer, patient education, numeracy, health risk communication, family communication, cascade screening

## Abstract

**Background/Objectives**: We aimed to test two different visual arrays for helping individuals accurately interpret colorectal cancer risk in Lynch syndrome. We also sought to examine factors associated with intention to share genetic test results with blood relatives. **Methods**: Participants were recruited through Amazon Mechanical Turk (July–August 2025). Participants completed an online REDCap survey evaluating genetic literacy/numeracy and risk perception (Tripartite Model of Risk Perception). Participants were randomized to a risk visualization—either a standard icon array or a sequential icon array intended to decrease cognitive load. Subsequently, participants were randomized to a communication choice scenario—a nondirective (choice) frame or an “enhanced choice” with a behavioral nudge based on the Theory of Planned Behavior. **Results**: Participants’ (n = 1041) estimation of colorectal cancer risk did not differ according to visualization type (icon array: 60.36 ± 15.86%, sequential array: 60.91 ± 15.73%, *p* = 0.58). Intention to share genetic testing outcomes with family members was not influenced by the behavioral nudge (*p* = 0.23). Neither risk estimation nor intent to communicate results to blood relatives were affected by individual perceived colorectal cancer risk, health literacy/numeracy, education, previous genetic testing, personal cancer history, or family cancer history (all *p* > 0.11 and *p* > 0.21, respectively). **Conclusions**: This study found that visual array type did not affect estimated cancer risk, and decision frame did not affect intention to share genetic testing results. Findings could inform the development of online approaches to expand decision support for hereditary cancer syndromes (Clinicaltrials.gov #NCT06994832).

## Simple Summary

Individuals with Lynch Syndrome are at increased risk for certain cancers, including colorectal cancer. Given the hereditary nature of Lynch Syndrome, understanding and communicating genetic risk within families is important to facilitate cascade carrier screening. This clinical trial tested two visual aids (arrays) to help people accurately interpret colorectal cancer risk and captured people’s intention to share genetic test results with family members. In the hypothetical genetic testing scenario, results indicate that visual array type did not affect how participants estimated cancer risk. Further, the way that cancer risk was presented (framed) in this study did not affect participants’ intention to communicate genetic cancer risk to family members.

## 1. Introduction

Lynch Syndrome (LS) is an autosomal dominant hereditary cancer predisposition syndrome [1,2]. The etiology of LS involves germline pathogenic variants in one of four key DNA mismatch repair genes (*MLH1*, *MSH2*, *MSH6*, *PMS2*) or deletions in *EPCAM* (*TACSTD1*) [1]. Notably, pathogenic variants underlying LS are relatively frequent (~1 in 280 individuals) and are one of the most common causes of inherited colorectal and endometrial cancers [3]. However, many individuals are unaware of their risk [4]. The U.S. Centers for Disease Control and Prevention considers LS a Tier 1 genetic condition, as widespread screening may have significant potential for positive public health impact [5]. Tier 1 classification supports the use of cascade carrier screening to identify at-risk individuals and mitigate morbidity and mortality [6,7,8]. A key barrier to more widespread cascade carrier screening is the shortage of individuals to support genetic testing (e.g., genetic counselors) and subsequent care [9].

Both clinicians and patients express interest in broader access to genetic risk information [10,11,12]. Particularly given the influence of perceived risk on health behaviors and decision making. In the context of LS, understanding perception is vital because family members who do not utilize genetic services may perceive their risk as too low to motivate preventative behaviors (i.e., colonoscopy), even when their objective risk is high [2]. Individuals do not typically assess their risk at face value; rather, they assess it based on belief systems, life experiences, and emotional states [13]. Understanding these factors allows us to move beyond a “one-size-fits-all” approach and create targeted strategies to better support how individuals perceive risk. Visual supports using pictographs (icon arrays) are a type of visual aid used in risk communication, and have long been considered one of the most effective methods for helping people accurately understand complex medical information [14,15,16].

A key goal of person-centered genomic healthcare is to support high-quality decisions (i.e., informed and aligned with values and preferences). Decision-making can be affected by a variety of factors. Cognitive load, the amount of mental effort required, can pose challenges for decision-making. Additionally, evidence suggests that people appraise risk differently. The Tripartite Model of Risk Perception (TRIRISK) identifies three types of risk appraisal: deliberative (rules-based/logical), affective (emotional worry/fear), and experiential (gut reactions/heuristic) perceptions of risk [17]. Further, how information is presented can influence decision-making, i.e., non-directive choice vs. framing with behavioral ‘nudges’ [18]. The primary aim of this study is to examine whether decreasing cognitive load on visual icon arrays would support more accurate risk appraisal for colorectal cancer in the context of LS. We also examine if framing decisions influenced intention to communicate genetic risk to blood relatives (enabling cascade carrier screening). We hypothesize that insights from this randomized trial could inform the development of asynchronous, digital decision support tools to expand cascade carrier screening for LS.

## 2. Materials and Methods

The study was approved by the Mass General Brigham IRB (Protocol #2025P000889) and the Boston College IRB (Protocol #26.0042). Adult participants were recruited through Amazon Mechanical Turk between 28 July and 18 August 2025 and completed an online REDCap [19,20] survey following opt-in electronic consent. Participants were provided with a description of colorectal cancer and LS, then completed a 30-item survey including sociodemographic information, measures of health literacy [21] and numeracy (4-item Berlin numeracy test) [22], and questions measuring deliberative (rules-based/logical), affective (emotional worry/fear), and experiential (gut reactions/heuristic) aspects of the TRIRISK model [17,23]. Additional details and steps taken to ensure data quality are described in Supplementary Materials.

**Figure 1:**
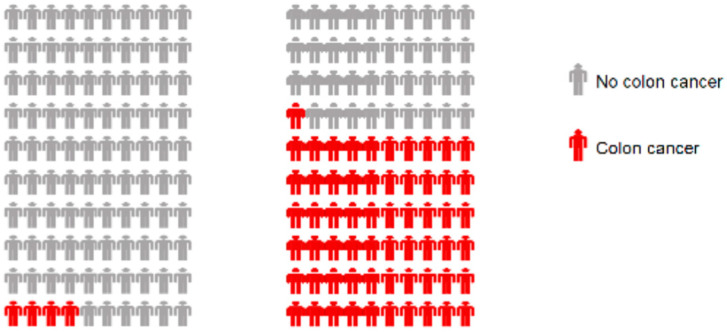
Icon arrays used in the study. The left array depicts 4% colorectal cancer risk for the general population. The right array shows 61% colorectal cancer risk for people with LS.

Participants were randomized using the REDCap randomization module into a 2 × 2 factorial design, consisting of two risk visualization formats and two choice array formats. The standard icon array (Figure 1) presents colorectal cancer risk for the general population (4%) side-by-side with Lynch syndrome (61%) [24]. The sequential array aims to decrease cognitive load by “chunking” information in three steps: (1) general population icon array; (2) Lynch syndrome icon array; (3) both arrays side-by-side. Participants provided an estimate of cancer risk based on the visualization to which they were randomized.

Using Ajzen’s Theory of Planned Behavior (TPB) [18] as a guiding theoretical model, we examined participants’ intention to share genetic test results with blood relatives. Briefly, the TPB posits that intention precedes action and three key mediators of intention are attitudes (i.e., positive/negative perceptions of the behavior), subjective norms (i.e., expectations of others), and perceived behavioral control (i.e., sense of agency and self-efficacy) [18]. Participants were randomized to the standard choice (i.e., non-directive) communication frame (CF) or “enhanced choice” (ECF) employing a ‘nudge’ drawing on the TPB subjective norms: “*Your healthcare provider tells you: It is important to share information with other family members who may be at risk. Many patients share their results with family members*”. A flow diagram detailing the study process is provided in Supplementary Materials (Figure S1).

### Analysis

We used *t*-tests to compare risk estimates across visualization conditions and *t*-tests to compare intention to communicate across choice frames. To estimate effect size, we calculated Cohen’s *d* to provide evidence for the direction of the effect as well as the 95% confidence intervals (CIs) of the difference between the two groups. To achieve 80% power to detect a small effect (Cohen’s *d* = 0.3), 175 participants per group are needed. We collected responses from approximately 500 participants per group, giving us 80% power to detect an effect of *d* = 0.18. Nevertheless, in cases for which the *p*-value was greater than 0.05, we calculated Bayes Factors (BF_10_). Briefly, BF_10_ is the Bayes factor in favor of the alternative hypothesis. Values less than 1.0 indicate evidence for the null hypothesis, with values from 0.10 to 0.33 indicating moderate evidence and values from 0.03 to 0.10 indicating strong evidence for the null hypothesis. We used linear regression to test direct effects of *a priori* confounding variables and potential indirect effects by mediating the manipulations of visualization and choice frame on risk estimates and intention, respectively. A *p*-value ≤ 0.05 was considered significant for all models, and Bonferroni correction was not used, given a priori predictions for each included confound.

For Likert scales that have 5 or more categories (as was used to measure TPB), it is reasonable to use a *t*-test if there is sufficient sample size (i.e., 500 per group), and the assumptions of normality and homoscedasticity were met for intention outcomes (verified using QQ-plots, boxplots, and stripplots). All criteria were achieved, thus validating the use of *t*-tests [25].

## 3. Results

In total, 1041 participants were included in the analysis. Participant characteristics are depicted in Table 1. Most participants were college-educated and self-identified as White and male. More than half of the participants reported having had prior genetic testing (either direct-to-consumer or in a clinical context). Prior to viewing a risk visualization, participants estimated their lifetime risk of getting colorectal cancer as 48.19% on average, whereas the general population risk is 1 in 13 males (4.3%), 1 in 25 females (3.9%) in 2024 [26].

### 3.1. Visual Presentation of Cancer Risk

Of those who responded to the question regarding cancer risk (n = 1001), participants randomized to the icon array (n = 515) (Figure 1) estimated the mean risk for colorectal cancer for people with LS to be 60.36 ± 15.86%. Participants randomized to the sequential array (n = 486) estimated risk to be 60.91 ± 15.73%. The two groups estimated the mean risk for the general population to be 46.71 ± 27.99% and 49.72 ± 7.92% for the icon array and sequential array, respectively. Regarding differences across groups, neither estimated LS risk (*p* = 0.58 d = 0.035, 95% CI [−2.51,1.42], BF = 0.08), nor general population risk (*p* = 0.09, d = 0.107, 95% CI [−6.46, 0.47], BF = 0.29) differed across presentation formats. Calculating the difference in estimated risk (LS minus the estimate for the general population) did not differ across presentation formats (*p* = 0.12, d = 0.098, 95% CI [−5.74, 0.66], BF = 0.23). The BF values indicate evidence for the null hypotheses. The *a priori* confounding variables (i.e., initial estimate of their risk for colorectal cancer, health literacy/numeracy, education, previous genetic testing, personal cancer history, family cancer history) did not affect risk estimation (all *p* > 0.11) and did not lead to differences in how visualization condition affected risk estimation (all *p* > 0.17 for the interactions of confounding variable by presentation format).

### 3.2. Intention to Communicate Risk

**Figure 2:**
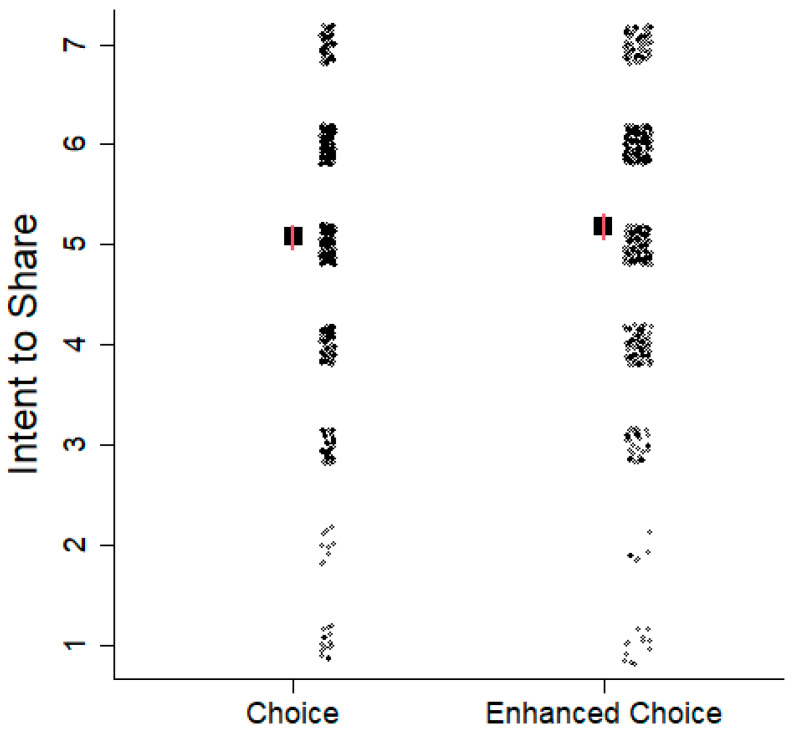
Intention to share hypothetical test results. Large squares depict mean intention to share genetic test results with family members (n = 1041). Red vertical bars show 95% CIs and are approximately the size of the marks. The small circles on the side show the intention to share with one circle, corresponding to one participant. Jitter was added to the horizontal axis to increase visibility.

Participants (n = 1041) were randomized to one of two decision frames—either a non-directive choice frame (n = 532) or the “enhanced choice” frame (n = 509) utilizing a TPB normative beliefs nudge. Regardless of decision frame, intention to share genetic test results with blood relatives was strikingly similar (*p* = 0.23, *d* = 0.076, BF = 0.14) (Figure 2). None of the potentially confounding variables (above) were associated with intention to share genetic test results with blood relatives (all *p* > 0.21).

### 3.3. Tripartite Risk and Theory of Planned Behavior

No significant associations were observed for items assessing TRIRISK (deliberative (rules-based/logical), affective (emotional worry/fear), and experiential (gut reactions/heuristic) perceptions of risk). Similarly, no significant associations were identified across items assessing TPB (attitudes, perceived norms, behavioral control).

## 4. Discussion

The study examined two different types of visual arrays (icon array vs. sequential array) to determine which visual aid helps individuals most accurately estimate colorectal cancer risk in LS. Prior to revealing the visualizations, we assessed participants’ perceived likelihood of developing colorectal cancer in their lifetime. The mean reported likelihood was 48 ± 25%, and only 26/1041 (2.5%) stated their lifetime risk as 3–4% (the risk for the general population [26]. Thus, we observed an unexpected baseline bias, such that perceived risk for the general population was inflated to approximately 12 times the actual risk. This finding raises an important dilemma for public health communication surrounding CRC. If the goal of public health communication is to correct misconceptions about CRC risk, this could potentially lower individuals’ concern for CRC. Alternatively, if the focus is to raise awareness of CRC and promote screening and mitigating risk through prevention and early detection, then less priority would be given to correcting the overestimation. Both groups accurately determined risk for LS, and attempts to decrease cognitive load via the sequential array did not improve the accuracy of estimated cancer risk. Thus, visual tools improved participants’ ability to accurately perceive risk. Drawing on principles of cognitive load theory, the highly educated population included in the study may have already been operating near optimal efficiency; thus, the intervention did not meaningfully reduce load further [27]. Suggesting that results may be influenced by a ceiling effect. Estimated risk values were already close to the true LS risk (61%), thus limiting observable increases.

As LS is a Tier 1 genetic condition, cascade carrier screening is highly relevant. With respect to sharing genetic information with family members, no differences were observed in intention to communicate results between the traditional non-directive choice frame compared to the “enhanced choice” frame that employed a nudge drawing on the TPB element normative behavior. Notably, no meaningful TRIRISK or TBP associations were identified. Several factors may have constrained observable relationships. First, measurement limitations may have reduced sensitivity to detect theoretically expected effects. Second, issues in operationalization may have contributed. We reduced complex theoretical domains to brief self-report measures and a single task context, which may not fully reflect the dynamic constructs described by TRIRISK and TPB frameworks. Additionally, it is possible that the results reflect limitations of the theories in this context; that is, the TRIRISK and TPB frameworks may be less predictive under the present task demands, sample characteristics, or decision environment. Furthermore, it is possible that our assumptions were overly reductionistic in assuming that the TRIRISK and TPB frameworks would readily elucidate study data. Finally, this study may reflect implementation boundaries, including the use of a hypothetical scenario, which may reduce the ability to detect meaningful effects.

While the results of this randomized trial could be considered largely null, the findings have relevance for initiatives to increase access to decisional support and thus cascade carrier screening. Research supports the cost-effectiveness of universal genetic testing (and cascade screening), following colorectal cancer diagnosis [28]. A significant bottleneck in cascade screening is the critical clinical genetics workforce shortage—even in high-income countries [9,29]. Some have proposed a “genetics first” approach, leveraging non-geneticist physicians to address the global shortage of geneticists [30]. Similarly, a recent systematic review has charted efforts to promote genomic practice among allied health professionals to expand services [31]. Notably, recent scoping reviews underscore that nursing is underutilized in genomic healthcare, highlighting how interprofessional collaboration between genetic counselors and nurses could expand genomic healthcare services—particularly in the context of hereditary cancer [32,33].

A central tenet of person-centered genomic healthcare is supporting individuals in high-quality decisions that are informed and aligned with values and preferences [34]. Similarly, supporting intrafamilial communication of risk is essential for enabling cascade carrier screening [35]. Recent work has highlighted the opportunity to scale decisional support and genomic services using digital platforms [36]. Such digital tools have been shown to decrease the number of face-to-face interactions with genetic counselors [37]. Advances in computing technology have propelled machine learning and artificial intelligence (AI), enabling large language models (LLMs) and chatbots for genetic testing decision support, as well as returning screening results [38,39]. Such chatbots have the added ability to communicate across language barriers and are acceptable to diverse populations [40,41]. There is an opportunity to support decision-making using AI/LLM and chatbots that are trained on a large corpus of robust and diverse data. Such an approach could elicit salient emotional drivers as part of the reflective process to support decisions that are aligned with emotional touchpoints, values, and preferences. AI-based clinical decision support systems are already used in cancer diagnosis and treatment [42]. However, such technology is limited by issues relating to transparency, bias, need for human validation and thus warrants careful ethical implementation [43].

There are several study limitations that merit mention. First, the study sample has high educational attainment and predominantly identifies as White and male, thereby limiting the generalizability of findings. Given the high educational attainment of the population, cognitive load effects may have been attenuated. This also should be taken into consideration when thinking about cascade screening and how uptake differs across sociodemographic groups [44]. It is plausible that a sequential array could be beneficial for individuals with more limited health literacy/numeracy. A robust body of literature supports that icon arrays are highly effective in populations with limited literacy and numeracy [13,45,46,47] as well as older adults [48]. While people with more educational attainment and higher health literacy/numeracy may perform well regardless of format, they still benefit from the added clarity of icon arrays [45,46]. Additionally, the simplicity of the task (i.e., single risk estimation) may have limited sensitivity to detect subtle differences. This work was drawn on prior work, and we acknowledge that this single frame has limited operationalization for the TPB. Future research may focus on more robust nudges (i.e., emotional framing), recognizing that patient choices are often influenced by emotions, habits, and cognitive biases [49,50]. Importantly, behavioral economics provides a useful framework for helping nurses support patients making complex decisions about genetic testing and genomics [51]. It is worthwhile to mention that the situation was hypothetical, so the emotional saliency of cancer is likely not the same as a real-life clinical example. We only developed one type of enhanced choice frame (drawing on normative beliefs). We did not examine the impact of other types of frames. Prior work has shown that different framing of genetic testing decisions increases the likelihood of opting for genetic testing, compared to non-directive choice [52]. In addition, emerging research in precision health and hyper-personalized care emphasizes that clinical decisions are increasingly informed by integrated biological, behavioral, and environmental data, rather than an isolated risk estimate [53]. It is possible that communicating results to blood relatives may be less susceptible to framing effects. In this study, we captured self-reported intention, which may not accurately reflect actual behavioral outcomes. The intervention was provided within the survey in a virtual format, so we were unable to control for participants’ attention and engagement with the materials. However, we did take several measures to remove patterned responses and responses that reflected inattention (Supplemental Materials). Finally, the survey captured intentions at a single point in time, and we do not know whether these intentions would translate into actual subsequent behavior.

## 5. Conclusions

Icon arrays are reliable and robust for conveying risk. Framing choice using a normative behavior nudge did not differentially affect intention to communicate genetic test results to blood relatives. Neither risk estimation nor intention to communicate risk was affected by individual colorectal cancer risk, health literacy/numeracy, education, previous genetic testing, personal cancer history, or family cancer history. Study findings could help inform the development of online decision support approaches, including AI/ML approaches, to increase access to genetic testing decision support.

## Figures and Tables

**Table 1:** Participant Self-reported Characteristics (n = 1041).

Age (Mean (sd))	35.54 (9.43)
Sex (Male (n = 1031))	695 (67.41%)
Race (n = 1016)	
White	933 (91.83%)
Asian	39 (3.84%)
Black or African American	28 (2.76%)
American Indian or Alaskan Native	10 (0.98%)
mixed	4 (0.39%)
Native Hawaiian or Pacific Islander	1 (0.10%)
prefer not to say	1 (0.10%)
Education (n = 1041)	
Bachelor’s Degree in College (4-year)	685 (65.80%)
Master’s Degree	244 (23.44%)
Some college but no degree	29 (2.79%)
Associate degree in college (2-year)	27 (2.59%)
Doctoral degree	21 (2.02%)
professional degree (JD, MD)	13 (1.25%)
high school graduate (high school diploma or equivalent, including GED)	21 (2.02%)
less than a high school degree	1 (0.01%)
positive personal history of cancer (n = 1041)	334 (32.94%)
positive family history of cancer (n = 1014)	585 (57.69%)
participated in genetic testing (n = 1014)	517 (50.99%)
Health literacy (n = 1028)	
Adequate	656 (63.81%)
Inadequate	372 (36.19%)

## Data Availability

Data supporting reported results can be found on the Harvard Dataverse https://dataverse.harvard.edu/dataset.xhtml?persistentId=doi:10.7910/DVN/T2JJHA.

## Supplementary Materials

The following supporting information can be downloaded at: https://www.mdpi.com/article/10.3390/cancers18091369/s1. Figure S1: Flow Diagram.

## Abbreviations

The following abbreviations are used in this manuscript:

TPB	Theory of Planned Behavior
CF	Choice frame
ECF	Enhanced choice frame
LS	Lynch Syndrome
TRIRISK	Tripartite Model of Risk Perception
LLM	Large language model
