# Supplementary figures and images for: Optimizing Risk Communication for Lynch Syndrome: Results of a Randomized Controlled Trial of Visual Arrays for Genetic Testing
Source: Cancers (Basel). 2026 Apr 25;18(9):1369. doi: 10.3390/cancers18091369 (PMC13163106; doi:10.3390/cancers18091369)

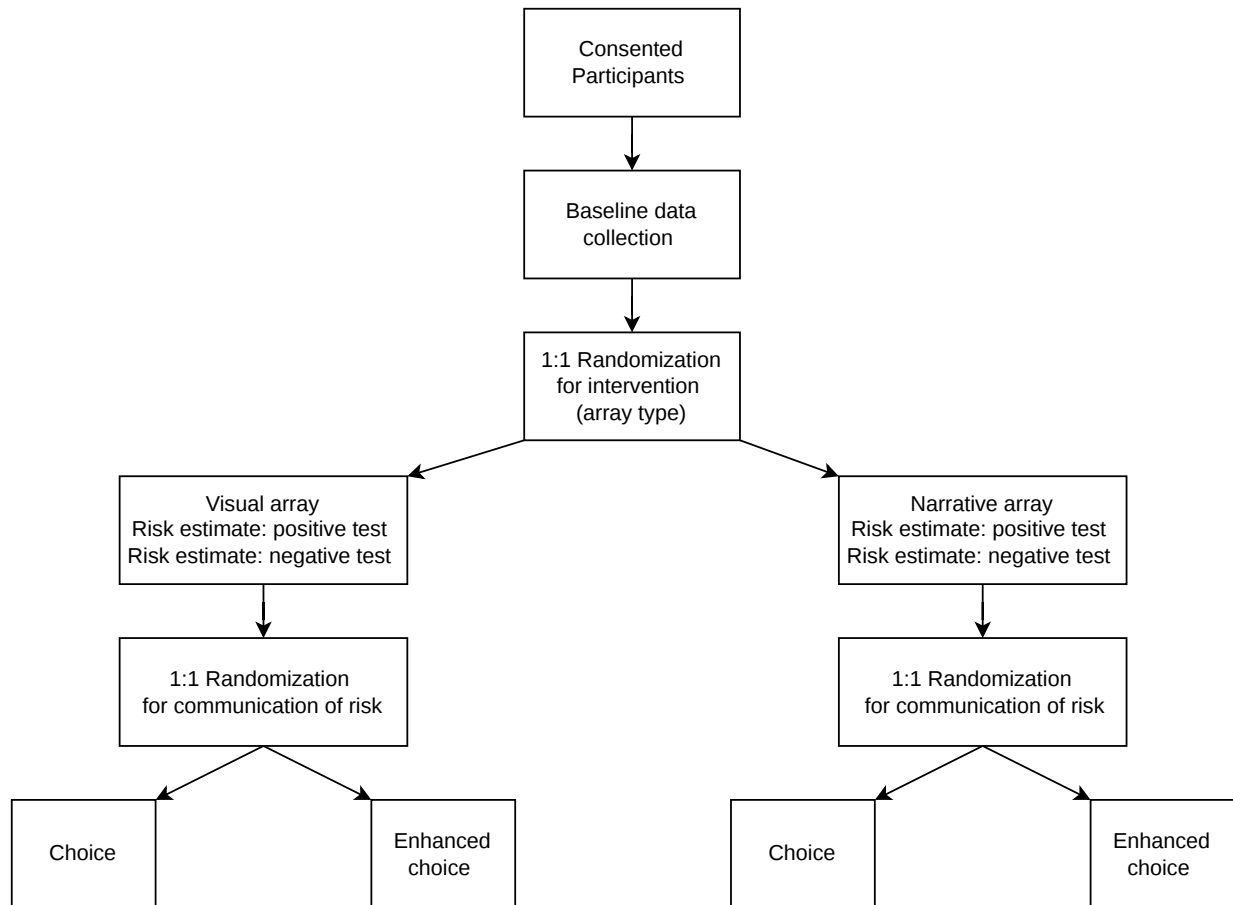

**Figure S1:** Flow Diagram

Supplement: Supplementary file 1 [file cancers-18-01369-s001.zip › cancers-4219902-supplementary.pdf]
